# Eco-Friendly Dye-Sensitized Solar Cells Based on Water-Electrolytes and Chlorophyll

**DOI:** 10.3390/ma14092150

**Published:** 2021-04-23

**Authors:** Ji-Hye Kim, Sung-Yoon Park, Dong-Hyuk Lim, So-Young Lim, Jonghoon Choi, Hyung-Jun Koo

**Affiliations:** 1 Department of New Energy Engineering, Seoul National University of Science & Technology, 232 Gongneung-ro, Nowon-gu, Seoul 01811, Korea; gh5289@naver.com; 2 Department of Chemical & Biomolecular Engineering, Seoul National University of Science & Technology, 232 Gongneung-ro, Nowon-gu, Seoul 01811, Korea; syoonpark@naver.com (S.-Y.P.); ljsldh5635@naver.com (D.-H.L.); thdud2306@naver.com (S.-Y.L.); 3 School of Integrative Engineering, Chung-Ang University, Seoul 06974, Korea; nanomed@cau.ac.kr

**Keywords:** dye-sensitized solar cells, aqueous electrolyte, chlorophyll, plasma treatment, eco-friendly devices

## Abstract

Organic solvents used for electrolytes of dye-sensitized solar cells (DSSCs) are generally not only toxic and explosive but also prone to leakage due to volatility and low surface tension. The representative dyes of DSSCs are ruthenium-complex molecules, which are expensive and require a complicated synthesis process. In this paper, the eco-friendly DSSCs were presented based on water-based electrolytes and a commercially available organic dye. The effect of aging time after the device fabrication and the electrolyte composition on the photovoltaic performance of the eco-friendly DSSCs were investigated. Plasma treatment of TiO_2_ was adopted to improve the dye adsorption as well as the wettability of the water-based electrolytes on TiO_2_. It turned out that the plasma treatment was an effective way of improving the photovoltaic performance of the eco-friendly DSSCs by increasing the efficiency by 3.4 times. For more eco-friendly DSSCs, the organic-synthetic dye was replaced by chlorophyll extracted from spinach. With the plasma treatment, the efficiency of the eco-friendly DSSCs based on water-electrolytes and chlorophyll was comparable to those of the previously reported chlorophyll-based DSSCs with non-aqueous electrolytes.

## 1. Introduction

Due to its advantages, such as a simple fabrication process, reasonable power conversion efficiency, and relatively low production cost [1,2], dye-sensitized solar cells (DSSC) have been actively researched for the past three decades. Even though the power conversion efficiency of the DSSCs reported is relatively low compared to the counterparts, e.g., perovskite and silicon solar cells [3,4,5], continuous efforts have still been made to improve the efficiency and replace the expensive noble metal catalysts with low-cost alternatives [6,7,8]. Furthermore, the enhanced photoconversion efficiency of DSSCs under ambient light was also achieved, suggesting the potential of DSSCs for indoor applications [9].

DSSCs adopted dye molecules to effectively absorb visible sunlight and generate electricity. Electrons in the dye were excited upon light absorption and then were injected into the high-surface-area-porous TiO_2_ film, thereby leaving holes in the dye. The oxidized dye molecules were regenerated by a redox reaction in electrolytes. DSSCs typically employed liquid electrolytes based on organic solvents, such as acetonitrile, methoxyacetonitrile, and 3-methoxypropionitrile. Even though these solvents enabled better performance, they were likely to be highly toxic, hazardous, or explosive and had an environmental impact [2,10]. Low viscosity, low surface tension, and high volatility of the organic solvents could also cause the leakage of the electrolytes [1,11]. Additionally, the representative dyes for DSSCs were ruthenium-complex molecules, which are costly and less eco-friendly. To overcome the disadvantages in the use of the organic solvents in electrolytes and the rare-transition metal-based dyes, research to apply water-based electrolytes [10,12] and organic synthetic or naturally-derived dyes [13,14] to DSSCs was conducted. However, the efficiency of such environmentally benign DSSCs was quite low and needs to be improved.

In this paper, we developed eco-friendly DSSCs based on aqueous electrolytes and organic synthetic dyes. The organic synthetic dye that is commercially available, was used as is without further modification to reduce the production cost and develop a more versatile procedure. The pristine dye was dissolved in the water-electrolyte, including iodine (I_2_) and potassium iodide (KI), to be kinetically adsorbed onto the TiO_2_ surface. The effects of aging time and plasma treatment of the TiO_2_ surface on the photovoltaic performance were investigated. For the fabrication of more eco-friendly DSSCs, the synthetic dye was replaced with naturally-derived chlorophyll dye extracted from spinach. The resulting efficiency of the DSSCs with chlorophyll was compared to that with the synthetic dye and the possible ways to improve the efficiency were discussed.

## 2. Materials and Methods

### 2.1. Materials

TiO_2_ paste (SC-HT040, particle size: 15–20 nm) was purchased from Sharechem Co. (Hwaseong, Korea). Chloroplatinic acid hexahydrate (>37.5%, Pt base), and eosin Y disodium salt (>85%) were purchased from Sigma-Aldrich (St. Louis, MO, USA). I_2_, KI, 2-propanol (IPA, 99.8%), and ethanol (95.0%) were obtained from Samchun Chemicals (Seoul, Korea). A poly(dimethylsiloxane), PDMS, elastomer kit (Sylgard 184 silicone elastomer base and curing agent) was purchased from Dow Corning Corp (Midland, TX, USA). PDMS was made by mixing the base and curing agent in a ratio of 10:1 by weight, degassing, and curing in an oven at 60 °C.

### 2.2. Fabrication of DSSCs with the Organic Synthetic Dye

Fluorine-doped tin oxide (FTO) glass substrates (Hanalin Tech, Seongnam, Korea) were washed by sonication in ethanol for 20 min. For a photoanode, the TiO_2_ paste was doctor-bladed on the FTO substrate with a thickness of 50 μm and then heated at 500 °C for 1 h in a furnace for sintering. For the Pt counter electrode, a solution of 0.005 M chloroplatinic acid hexahydrate in IPA was cast on an FTO glass with a size of 25 mm × 13 mm and was heated at 400 °C for 1 h in a furnace. The 100 μm-thick PDMS spacer was sandwiched between the anode and cathode for electrolyte filling. For the aqueous dye-electrolyte solution, 0.4 M KI, 0.02 M I_2_, and 0.005 M eosin Y salt were dissolved in deionized (DI) water. The plasma treatment (100 W, 50 kHz) of the TiO_2_ photoanodes was performed for 5 min to 60 min with a plasma system (CUTE, Femto Science Inc., Gyeonggi-do, Korea) before filling the electrolyte.

### 2.3. Fabrication of Chlorophyll Based DSSCs

Spinach leaves were washed with cold water, crushed, and ground in a mortar. 100 mL of ethanol was added to 10 g of spinach and then was stored in an oven at 60 °C for 30 min for chlorophyll extraction. The dark-green-colored solution obtained was filtered and centrifuged at 3000 rpm for 10 min. The resulting supernatant was used as the chlorophyll extract. The extract and DI water were mixed in a ratio of 2:1 by the volume for dyeing solution. A TiO_2_ photoanode was immersed in the solution for 24 h for chlorophyll adsorption. After washing with DI water, the photoanode was assembled with the Pt counter electrode and the PDMS spacer. Finally, the aqueous electrolytes with 0.4 M KI and 0.02 M I_2_ were injected. The plasma treatment (100 W, 50 kHz) of the TiO_2_ photoanodes was performed for 60 min before the chlorophyll adsorption process.

### 2.4. Photovoltaic Measurement

The current density–voltage (*J–V*) curves of the DSSCs were measured using a Keithley 2400 source meter under the illumination of the simulated solar light (100 mW/cm^2^, AM 1.5 G, Sol3A, Newport, Irvine, CA USA). The solar simulator was calibrated with an encapsulated reference silicon solar cell certified by the Newport Cop. PV Lab (California, USA). The active area was ~0.25 cm^2^.

## 3. Results and Discussion

**Figure 1:**
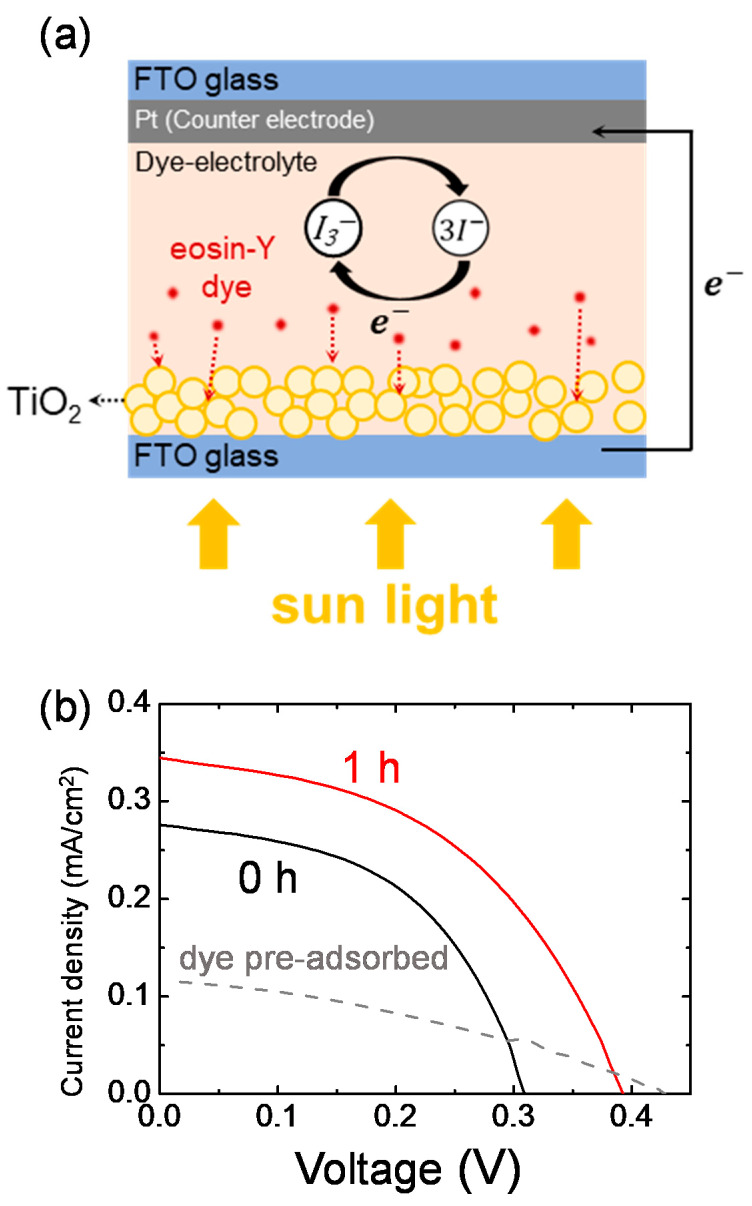
(**a**) The structure of eco-friendly DSSCs. (**b**) Density–voltage (*J–V*) graphs of the eco-friendly DSSCs at aging time of 0 h and 1 h. The concentration of the dye-electrolyte was 5 mM eosin Y dye, 0.4 M KI, and 0.02 M I_2_. The dotted line is the *J–V* graph of the DSSCs where the eosin Y dye was pre-adsorbed onto TiO_2_ photoanode.

Figure 1a shows the structure of the eco-friendly DSSC. The commercial eosin Y dye itself was not effectively adsorbed onto TiO_2_ and was easily desorbed during washing after the dyeing step. Instead of the typical DSSCs fabrication process, we introduced the dye into the electrolyte, i.e., dye-electrolyte, so that the dye molecules were adsorbed onto TiO_2_ via adsorption equilibrium. Figure 1b and Table 1 compare the photovoltaic characteristics of the eco-friendly DSSCs with the dye-electrolyte to that of the DSSCs with the pre-adsorbed dye. As fabricated, the eco-friendly DSSCs showed 0.044% efficiency, which was much higher than that of the DSSCs with the pre-adsorbed dye. After 1 h of aging at room temperature, the efficiency of the eco-friendly DSSCs increased by 45%, from 0.044% to 0.064%. This was possible because it took time for the aqueous electrolytes to fully wet the TiO_2_ surface or for the adsorption of dye molecules onto the TiO_2_ to be equilibrated. As the dye-electrolyte permeated onto the porous TiO_2_, the contact area between dye, electrolyte, and TiO_2_ increased, and the series resistance in DSSCs decreased, resulting in an increase in efficiency [15]. It was found that, for the eco-friendly DSSCs, it could be more efficient to include dyes in the aqueous electrolytes instead of the pre-adsorption of dyes onto TiO_2_, and an appropriate aging process would be required for higher efficiency.

**Figure 2:**
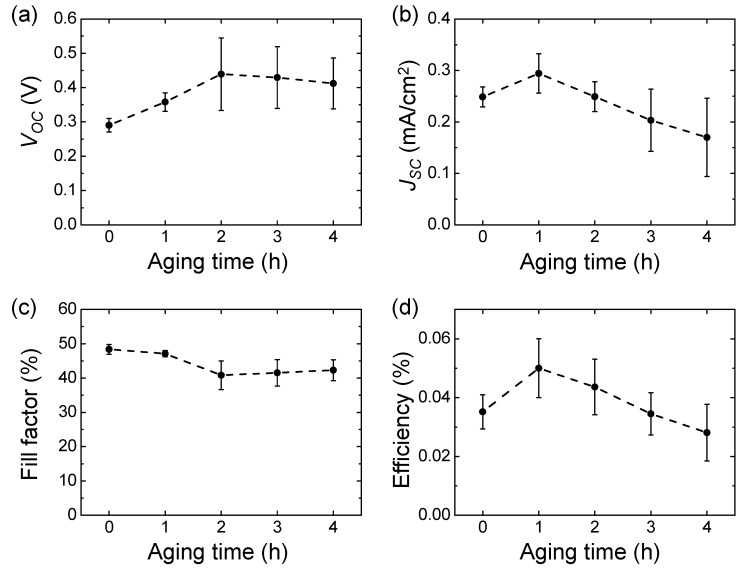
Photovoltaic characteristics of the eco-friendly DSSCs as a function of aging time: (**a**) *V_OC_*, (**b**) *J_SC_*, (**c**) fill factor, and (**d**) efficiency. The concentration of the dye-electrolyte was 5 mM eosin Y dye, 0.4 M KI, and 0.02 M I_2_.

Figure 2 shows the changes in *V_OC_*, *J_SC_*, fill factor, and efficiency of eco-friendly DSSCs depending on aging time. The *V_OC_* and fill factor did not change significantly over time, while *J_SC_* and the resulting efficiency were highest at 1 h of aging time and then gradually decreased. The possible reasons for the decrease in *J_SC_* are the following: (1) dye degradation [16,17,18], (2) dye detachment from the TiO_2_ surface into the electrolyte by strong adsorption of water molecules onto the TiO_2_ [19,20], and (3) recombination by contact of the dye-free TiO_2_ surface with aqueous electrolytes [15]. A further experiment is now underway to improve the stability of the eco-friendly DSSCs by selecting more durable, commercially available organic dyes.

**Figure 3:**
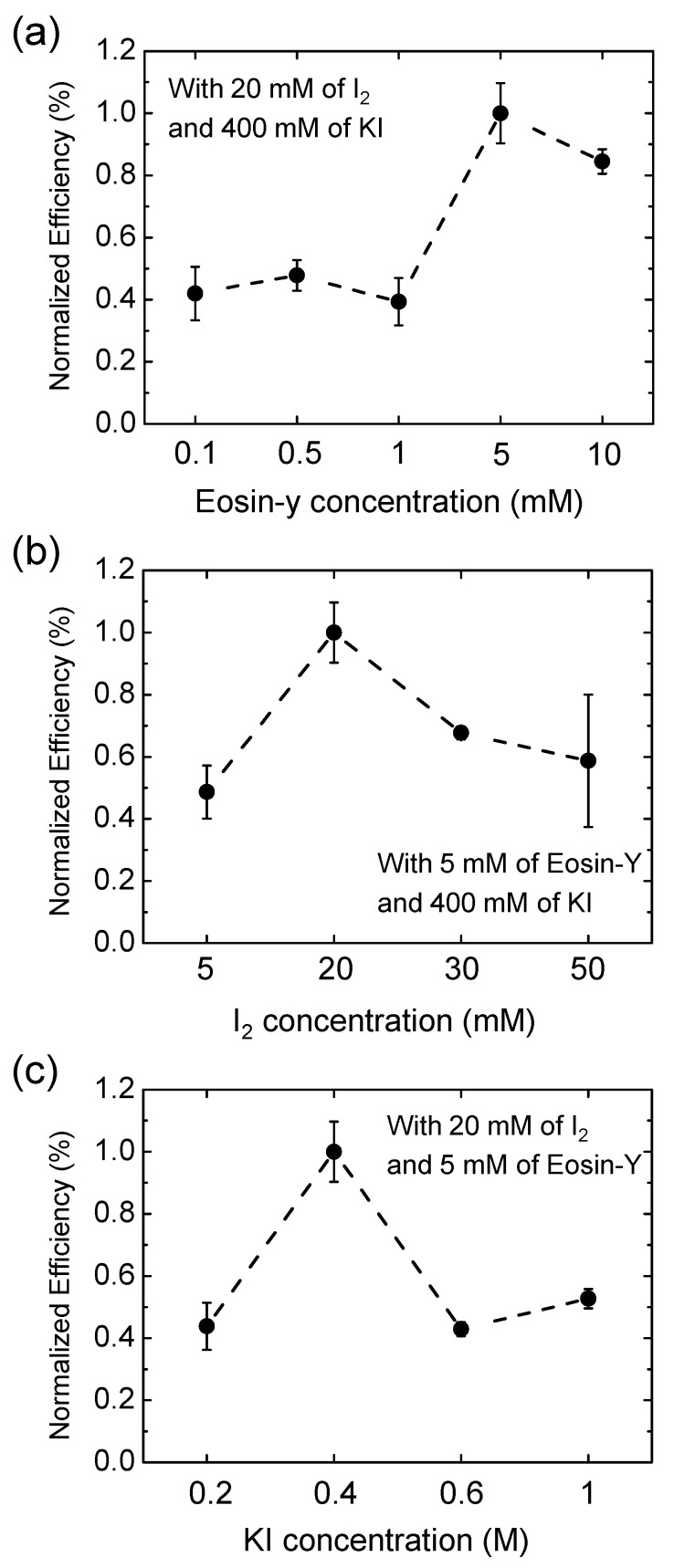
Effect of dye-electrolyte concentration on efficiency according to (**a**) concentration of eosin-Y dye, (**b**) concentration of I_2_, and (**c**) concentration of KI. The efficiency values were normalized based on that with the dye-electrolyte including 5 mM eosin Y dye, 0.4 M KI, and 0.02 M I_2_.

The dye-electrolyte concentration in the eco-friendly DSSCs was optimized by comparing the efficiency depending on the concentration of the eosin Y dye, I_2_, and KI (Figure 3). When the concentration of eosin Y was less than 1 mM, there was no significant change in the efficiency. The efficiency was highest at 5 mM of eosin Y and slightly decreased at 10 mM. Similarly, the efficiency was highest at 20 mM of I_2_ and 0.4 M of KI. Thus, the photovoltaic efficiency of the eco-friendly DSSCs was strongly affected by the concentration of the dye-electrolyte, and the resulting optimal composition was 5 mM of eosin Y dye, 20 mM of I_2_, and 0.4 M of KI.

**Figure 4:**
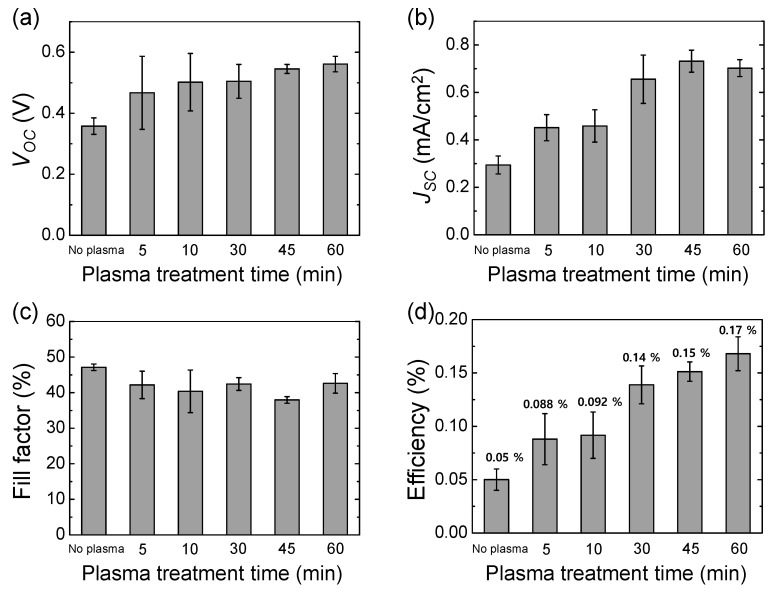
Effect of the plasma treatment of TiO_2_ on photovoltaic characteristics of the eco-friendly DSSCs: (**a**) *V_OC_*, (**b**) *J_SC_*, (**c**) fill factor and (**d**) efficiency. The atmospheric air plasma treatment was used. The concentration of the dye-electrolyte was 5 mM eosin Y dye, 0.4 M KI, and 0.02 M I_2_.

It was reported that the plasma treatment of TiO_2_ could enhance hydrophilicity [21,22], surface roughness and reactivity [23,24], and the reduction of oxygen vacancies [22,25,26,27]. We hypothesized that these factors would improve the dye adsorption onto TiO_2_ and the affinity of an interface of TiO_2_ and the water-based electrolyte. Figure 4 and Table 2 show the effects of the plasma treatment of TiO_2_ on the photovoltaic performance of the eco-friendly DSSCs. As the plasma treatment time increased, the *V_OC_* and *J_SC_* were significantly improved until 45 min of plasma treatment, while the fill factor hardly changed between 40–50%. As a result, the average efficiency increased by ~3.4 times from 0.05 to 0.17% via 1 h of plasma treatment of TiO_2_. Thus, it turned out that the plasma treatment of TiO_2_ was an effective way of improving the photovoltaic performance of the eco-friendly DSSCs.

**Figure 5:**
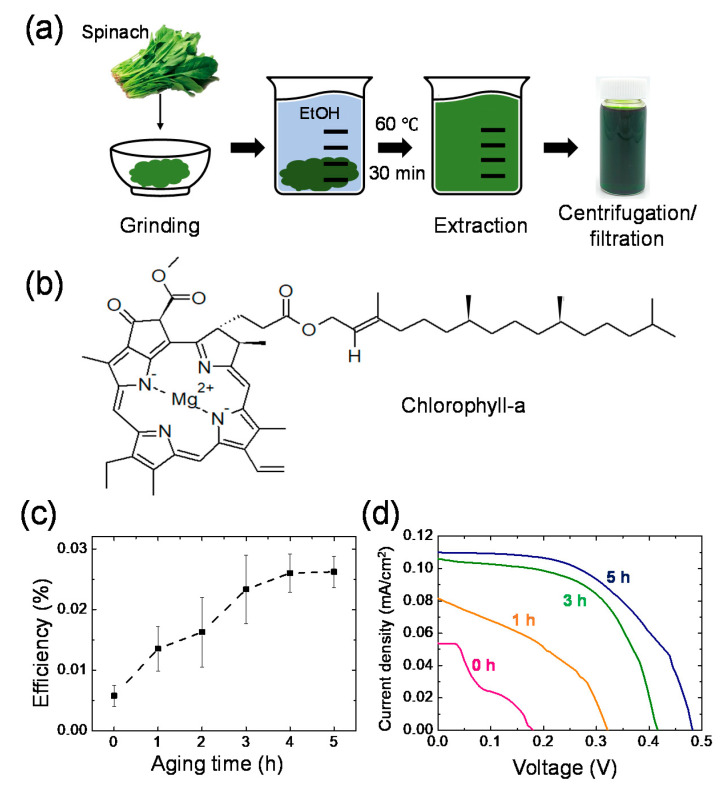
(**a**) Process of chlorophyll extraction from spinach. (**b**) Molecular structure of chlorophyll-a. (**c**) Change in the efficiency of the eco-friendly DSSCs with chlorophyll depending on the aging time and (**d**) corresponding *J–V* graphs.

As the next step toward further eco-friendly DSSCs, the organic synthetic dye was replaced with chlorophyll derived from natural spinach leaves. The extraction process to obtain the chlorophyll stock solution is shown in Figure 5a. Due to its long hydrocarbon chain, as shown in Figure 5b, chlorophyll was barely soluble in aqueous electrolytes. Instead, TiO_2_ was stained for 24 h in the solution where the chlorophyll stock solution and DI water were mixed at the ratio of 2:1 (*v*/*v*) [28,29]. The TiO_2_ photoanode stained by the spinach chlorophyll was assembled with the spacer and the Pt counter electrode, followed by adding the aqueous KI/I_2_ electrolytes. Figure 5c,d shows the photovoltaic characteristics of the eco-friendly DSSCs with chlorophyll as a function of aging time. The efficiency of the DSSCs gradually increased with time and stabilized after 3 h of aging time. The resulting efficiency after the aging step was ~0.026%, which was higher than that of the DSSCs with pre-adsorbed eosin Y, as discussed in Table 1.

**Figure 6:**
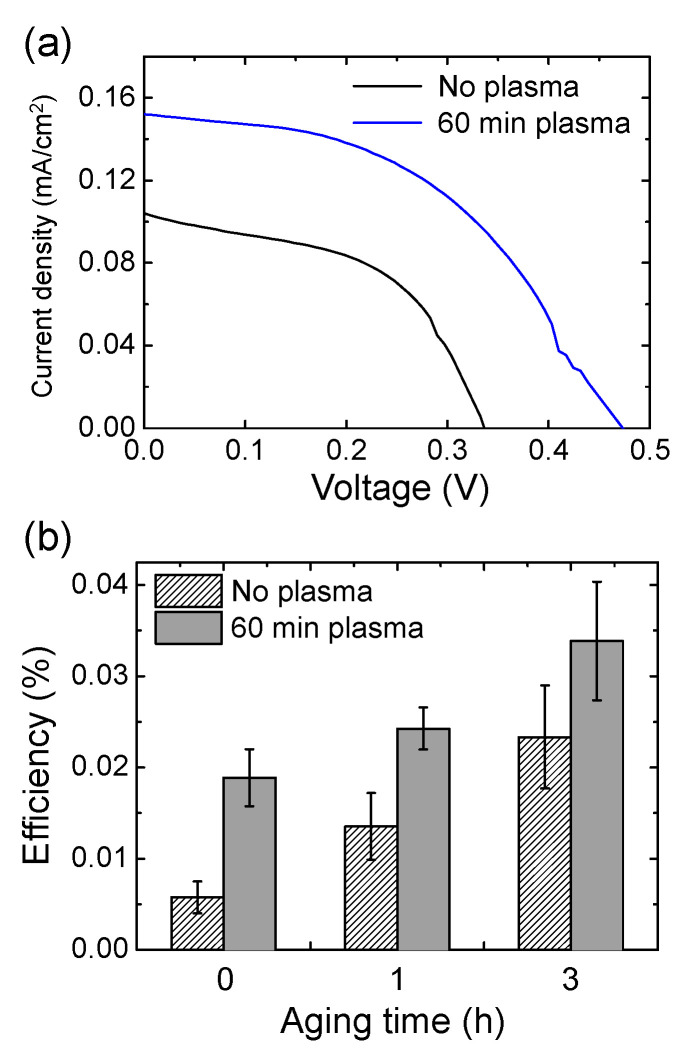
(**a**) *J–V* curve graphs of the eco-friendly DSSCs with chlorophyll with and without plasma treatment for 60 min at 3 h of aging time. (**b**) Changes in the efficiency of DSSC with and without plasma treatment for 60 min according to aging time.

We investigated the effect of the plasma treatment of the TiO_2_ photoanode on the photovoltaic performance of the eco-friendly DSSCs based on chlorophyll. It was also reported that chlorophyll was better adsorbed onto the plasma-treated TiO_2_ surface due to the reduction of the oxygen vacancies [25,30]. The TiO_2_ photoanode of the eco-friendly DSSCs was treated with air plasma for 60 min before chlorophyll staining. Figure 6 and Table 3 compare the photovoltaic characteristics of the chlorophyll-based DSSCs with and without the plasma treatment. Both *V_OC_* and *J_SC_* increased after the plasma treatment for 60 min. As a result, when compared at 3 h of aging time, the plasma treatment improved the efficiency by ~50%. Notably, the resulting efficiency was comparable to those of the previously reported chlorophyll-based DSSCs with non-aqueous electrolytes [31,32,33]. The efficiency was still low, even compared to the eosin-Y-based DSSCs in Figure 4d. This was largely due to the low current density and needed to be significantly improved for practical use. According to the literature [34], 10 g of spinach contains 6.91 mg of chlorophyll. In this study, if we assume that all of the chlorophyll molecules in spinach were completely extracted, the maximum molar concentration of the chlorophyll staining solution was ~0.052 mM. Therefore, the actual concentration should be relatively low compared to the typical concentration for dye staining of DSSCs [35,36,37]. The efficiency of the eco-friendly DSSCs could be further improved by optimizing the conditions of the chlorophyll staining and the plasma treatment.

## 4. Conclusions

In conclusion, the eco-friendly DSSCs were fabricated by employing the water-based electrolyte and the commercial organic dye of eosin Y. The commercial dye without any further modification was introduced into the water-electrolytes to deal with the issue of poor adsorption of eosin Y onto TiO_2_. The effects of the aging time and the composition of the dye-electrolyte on the photovoltaic performance of the eco-friendly DSSCs were investigated. To improve the dye adsorption and wettability of the water-based electrolyte, the surface of the TiO_2_ photoanode was treated by air plasma. It turns out that the plasma treatment was highly effective. The photovoltaic efficiency of the eco-friendly DSSCs increased by ~3.4 times after the plasma treatment, compared to that without the plasma treatment. For more eco-friendly DSSCs, the organic synthetic dye was replaced by the naturally-derived chlorophyll. Finally, the eco-friendly DSSCs based on the chlorophyll photosensitizer were fabricated. The resulting efficiency with the plasma treatment was comparable to those of the chlorophyll-based DSSCs with non-aqueous electrolytes. Even though many issues still need to be solved, such as low photovoltaic efficiency, expensive Pt catalyst, etc., we believe that such an eco-friendly DSSCs based on aqueous electrolytes and natural photosensitizers could be the suitable energy device structure with a minimum environmental footprint in the future.

## Figures and Tables

**Table 1:** Photovoltaic performances of water-based dye-sensitized solar cells (DSSCs) at different aging times, compared with the DSSCs with pre-adsorbed dye.

State of Dye	Aging Time (h)	*V_OC_* (V)	*J_SC_* (mA/cm^2^)	Fill Factor (%)	Efficiency (%)
Dye in electrolyte (dye-electrolyte)	0	0.31	0.28	50.3	0.044
	1	0.39	0.35	47.1	0.064
Dye pre-adsorbed	-	0.43	0.12	34.2	0.017

**Table 2:** Photovoltaic performance parameters of the eco-friendly DSSCs with and without plasma treatment at 1 h of aging time.

Plasma Treatment	*V_OC_* (V)	*J_SC_* (mA/cm^2^)	Fill Factor (%)	Efficiency (%)
Without plasma	0.35	0.29	47.1	0.05
With plasma for 60 min	0.56	0.70	42.6	0.17

**Table 3:** Photovoltaic performance parameters of the chlorophyll-based DSSCs with and without plasma treatment at 3 h of aging time.

Plasma Treatment	*V_OC_* (V)	*J_SC_* (mA/cm^2^)	Fill Factor (%)	Efficiency (%)
Without plasma	0.46	0.089	56.4	0.023
With plasma for 60 min	0.46	0.14	52.4	0.033

## Data Availability

The data presented in this study are available on request from the corresponding author.
